# Bioaccumulation and Bioremediation of Heavy Metals in Fishes—A Review

**DOI:** 10.3390/toxics11060510

**Published:** 2023-06-06

**Authors:** Farhan Jamil Emon, Md Fazle Rohani, Nusrat Sumaiya, Mst Fatema Tuj Jannat, Yeasmin Akter, Md Shahjahan, Zulhisyam Abdul Kari, Albaris B. Tahiluddin, Khang Wen Goh

**Affiliations:** 1 Laboratory of Fish Ecophysiology, Department of Fisheries Management, Bangladesh Agricultural University, Mymensingh 2202, Bangladesh; emon.1706069@bau.edu.bd (F.J.E.); sumaiya.1706047@bau.edu.bd (N.S.); jannat.limpa@gmail.com (M.F.T.J.); 2 Department of Aquaculture, Bangladesh Agricultural University, Mymensingh 2202, Bangladesh; rohani_aq@bau.edu.bd; 3 Department of Applied Chemistry and Chemical Engineering, Noakhali Science and Technology University, Noakhali 3814, Bangladesh; yeasmin.acce@nstu.edu.bd; 4 Department of Agricultural Sciences, Faculty of Agro-Based Industry, Universiti Malaysia Kelantan, Jeli Campus, Jeli 17600, Malaysia; zulhisyam.a@umk.edu.my; 5 Advanced Livestock and Aquaculture Research Group, Faculty of Agro-Based Industry, Universiti Malaysia Kelantan, Jeli Campus, Jeli 17600, Malaysia; 6 College of Fisheries, Mindanao State University-Tawi-Tawi College of Technology and Oceanography, Sanga-Sanga, Bongao 7500, Philippines; albarist20@gmail.com; 7 Faculty of Data Science and Information Technology, INTI International University, Nilai 71800, Malaysia

**Keywords:** pollution, bioaccumulation, bioremediation, phytoremediation, trace elements, sustainable aquaculture

## Abstract

Heavy metals, the most potent contaminants of the environment, are discharged into the aquatic ecosystems through the effluents of several industries, resulting in serious aquatic pollution. This type of severe heavy metal contamination in aquaculture systems has attracted great attention throughout the world. These toxic heavy metals are transmitted into the food chain through their bioaccumulation in different tissues of aquatic species and have aroused serious public health concerns. Heavy metal toxicity negatively affects the growth, reproduction, and physiology of fish, which is threatening the sustainable development of the aquaculture sector. Recently, several techniques, such as adsorption, physio-biochemical, molecular, and phytoremediation mechanisms have been successfully applied to reduce the toxicants in the environment. Microorganisms, especially several bacterial species, play a key role in this bioremediation process. In this context, the present review summarizes the bioaccumulation of different heavy metals into fishes, their toxic effects, and possible bioremediation techniques to protect the fishes from heavy metal contamination. Additionally, this paper discusses existing strategies to bioremediate heavy metals from aquatic ecosystems and the scope of genetic and molecular approaches for the effective bioremediation of heavy metals.

## 1. Introduction

Heavy metal contamination in aquatic water bodies is a major concern that has a serious impact on the associated organisms, especially fish [1,2,3]. Heavy metals naturally exist in the environment, but excessive application in different industries for several purposes has significantly altered the ecological system [4] by the excessive discharge of these metals into the soil and aquatic systems [5,6]. Generally, anthropogenic activities, such as the culture of crop foods, erosion from agricultural fields, and the discharge of industrial and household wastes, are considered main sources of heavy metals in aquatic systems [7,8]. Once heavy metals enter the aquatic systems, they are dissolved in the water and easily accumulate in the different parts of aquatic living organisms, including fish, and subsequently enter into consumers of these contaminated fish [9]. The bioaccumulation of heavy metals in fish causes several complications for fish health and their physiological activities [10]. The severity of metal toxicity (carcinogenic, teratogenic, and mutagenic) varies significantly with the fish species, the level of the metals, and the period of exposure [11]. Aquatic organisms, including fish, can be contaminated with heavy metals sourced from both the water as well as sediments of the aquatic ecosystems [12]. Heavy metal-mediated toxicity adversely damages the nervous system of fish, which negatively disrupts the interaction of fish with the surrounding environment [13]. The uncontrolled use and accumulation of these metals have become an important issue of health concern as most do not have the ability to break down into nontoxic states and, hence, have destructive effects on human health as well as aquatic organisms [14,15,16]. Heavy metal contamination negatively affects the growth and reproductive activity of fish by lowering their gonadosomatic index (GSI), fecundity, fertilization, and hatching rate [17,18,19,20,21,22]. Moreover, the toxicity of heavy metals disrupts the normal growth and progress of fish embryos and larvae [3,23,24,25,26]. Although various metals are essential for living organisms [27,28], most are very dangerous, even in a very small amount [29,30]. Moreover, some of the metals, namely arsenic (As), cadmium (Cd), copper (Cu), chromium (Cr), lead (Pb), mercury (Hg), nickel (Ni), selenium (Se), zinc (Zn), etc., are not only highly toxic but also result in carcinogenicity and mutagenicity [31,32,33,34].

Although several physico-chemical methods are available to remove these toxic heavy metals, most of these techniques seem ineffective when the concentrations of metals are lower than 100 mg/L [35]. As many heavy metals are soluble in water and dissolve in contaminated water, it is very difficult to separate them through the application of physical methods [36]. In this situation, biological methods such as bioremediation can be an attractive solution to rectify the natural condition of the environment from heavy metal contamination [4]. Bioremediation is considered one of the most environmentally friendly and sustainable ways to reduce several aquatic contaminations, which plays a significant role in improving the production of associated aquaculture systems [37,38]. Generally, the bioremediation process is very effective in reducing the toxicity of heavy metals by converting them into less harmful forms with the help of either microbe [39,40] or their enzymes to lessen the contamination [41]. This is considered an ecofriendly and cost-effective method to revitalize the contaminated environment [36,42]. Microorganisms with catabolic potentiality or their derived substances, including enzymes and biological surfactants, are an innovative strategy to facilitate remediation efficiency [43,44,45]. Microorganisms have the capability to synthesize metals, and this is widely used as a green approach to reducing metal-associated contamination [46]. Synthesis of nanomaterials through different microorganisms has been widely employed in wastewater treatment throughout the world [47,48,49,50,51]. These nanoparticles synthesized by microorganisms can effectively remove and recycle heavy metals from heavy metal-contaminated aquatic systems without changing their stability [52]. Several studies reported that genetically transformed microorganisms could efficiently enhance the adsorption ability and be successfully used for the remediation process [53,54]. The remediation capacity of microorganisms can be enhanced with the combination of several modifications, including biochar, biosurfactants, compost, and inorganic nutrients [55,56,57]. Moreover, several modern approaches in microbe-intervened biotechnologies, such as rhizoremediation [58,59], genetically engineered organisms [4,60,61,62,63], and nanotechnological intervention in microbial bioremediation [64,65,66,67], have been widely applied in the bioremediation of several toxic heavy metals from the environment. Despite the destructive impact of heavy metal bioaccumulation in fish, no comprehensive information is available on the remediation of these toxic heavy metals in fishes. Therefore, the current review summarizes the recent information regarding bioaccumulation and developments in bioremediation techniques of heavy metals in fishes.

## 2. Bioaccumulation of Heavy Metals in Different Tissues of Fishes

**Figure 1:**
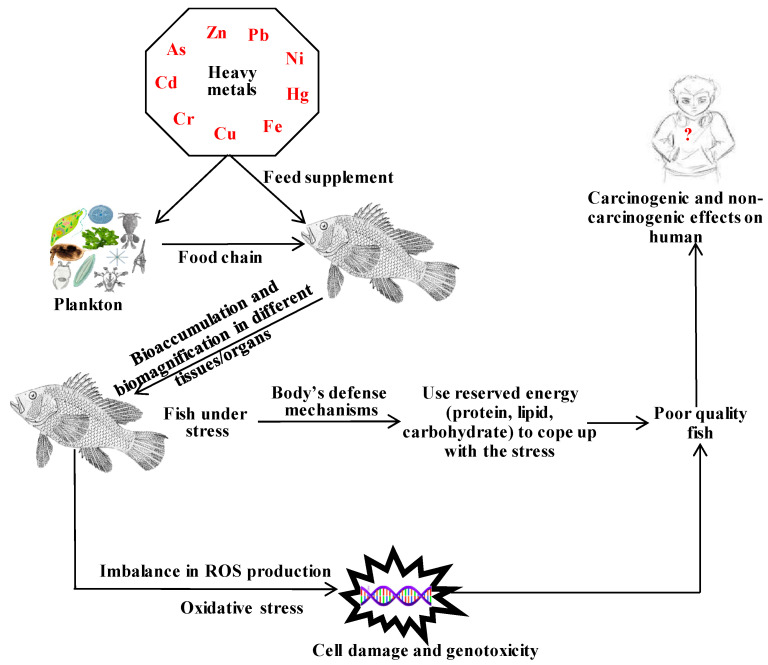
Routes of heavy metal accumulation in fish; ROS, reactive oxygen species.

Bioaccumulation assessment is one of the important indications for monitoring the geochemical cycle of heavy metals in the aquatic ecosystem. Toxic effects and oxidation of heavy metals vary with their forms and metal types, respectively. Chromium (Cr) generally exists in six different oxidative forms (+1 to +6), among which hexavalent Cr exerts destructive effects in fish [68]. Fish in heavy metal-contaminated aquatic systems pose a serious threat as fish accumulate metals through several important body tissues (gills, liver, kidney, skin, muscle, etc.), which are clearly illustrated in Figure 1. Fish require more energy, which is sourced from reserved nutrients, including protein, fats, and carbohydrates, to acclimate themselves in this stressed condition. Some of the metals (As, Cd, Cr, Cu, Fe, Hg, Ni, Pb, Zn) have redox potentiality, and they react to produce reactive oxygen species (ROS) that play an important role in maintaining a certain physiology in fish. ROS acts as an indicator of state oxidative stress that restricts the activity of cells through degrading protein, lipids, and DNA. Heavy metals bioaccumulate into different aquatic organisms through the food cycle and cause serious human health issues upon consumption of these contaminated fish [69,70,71,72,73]. Bioaccumulation of heavy metals in different fish organs is presented in Table 1, and different toxic effects of heavy metals in fish are demonstrated in Table 2.

### 2.1. As

As is one of the most toxic heavy metals which pollute aquatic water bodies by means of various natural as well as man-made actions [74]. It has been reported that inorganic As resulted in more toxic than organic forms [75,76]. As accumulates in different organs of fish (Table 1) at different rates [77,78]. It has been revealed that the highest amount of As (10.04 ± 2.99 μg/g) accumulated in the liver, whereas the lowest (3.74 ± 3.38 μg/g) was observed in muscle after 20 days of exposure by *Oreochromis niloticus* [57]. Several studies reported that As exposure caused various negative impacts on fish, such as growth and production reduction, hemato-biochemical changes, hormonal dysfunctions, histopathological anomalies, embryonic and larval development retardation, and other diseases [79,80,81,82,83]. Moreover, As toxicity significantly affected the hematology and immunology of several fish [84,85,86,87]. A high dose of As resulted in high mucus release, abnormal swimming, and loss of balance in *Anabas testudineus* and *Danio rerio* [88,89]. As stimulated the formation of apoptosis, micronuclei, and several cellular and nuclear abnormalities in erythrocytes of fish [82]. As induced several cytotoxicities and genotoxicities in medaka, *Oryzias latipes* [90]. Moreover, As contamination disrupted the reproductive activities of fish by inhibiting the gametogenesis process and, thus, negatively affecting sperm and ovum quality as well as quantity, fertilization, and hatching success [91,92,93].

### 2.2. Cd

Cd is very toxic and carcinogenic to humans and several animals, including fish. According to the Agency for Toxic Substances and Disease Registry of the United States, this metal ranks as the seventh most hazardous agent [94]. Several studies reported that the aquatic environment is significantly contaminated with Cd [95,96,97]. Assimilation and bioaccumulation of this toxic metal has occurred in a wide range of aquatic species (Table 1). Cd toxicity has resulted in the dysfunction of several important organs of fish, such as the liver, kidney, and gills, which affects the physiology of fish and hampers their growth [98,99,100]. Additionally, Cd alters the hematological indices by disturbing iron metabolism and creating anemic conditions [101,102]. Cd causes inhibition of antioxidant enzymes, inducing lipid peroxidation in animals [103,104]. Moreover, Cd toxicity negatively affects the reproductive performance of fish by shrinking the lobules of sperm, creating fibrosis in testis and lowering sperm motility and viability [19,76,105,106,107].

### 2.3. Cr

Cr is a ubiquitous metal that deteriorates the environment quality sourced from different types of industries [108,109]. Several studies reported the bioaccumulation of Cr in the different organs (Table 1) of *Cyprinus carpio* [110,111], *Carassius auratus* [112], *O. aureus* [113], and *Cirrhinus mrigala* [109]. Cr toxicity disturbs the physiological functions of fish and results in various allergic as well as organ-system failure [5,114,115,116,117]. Additionally, Cr toxicity significantly alters the protein, lipid, and glycogen content in the muscle, liver, and gills of *Labeo rohita* [118] and *C. mrigala* [119], causes hepatic stress in *C. auratus* [120], disturbs the functions of important organs (liver, kidney) of *Ctenopharyngodon idella* [121], and causes abnormal functions of the endocrine system of several freshwater fish species [68]. Cr was found to alter the blood profile, resulting in cellular and nuclear abnormalities of *Pangasianodon hypophthalmus* [1,117]. Several studies reported that high Cr levels in fish diets significantly decreased the growth and feed utilization of different fish species [122]. Moreover, chronic Cr exposure resulted in complexities in the reproduction of fish by lowering spawning success [123,124], deforming testis [19], decreasing sperm motility [105], and hampering the formation of oocytes [125].

### 2.4. Cu

Cu is a major contaminant of aquatic systems that results in stressful conditions for the aquatic organisms and significantly hampers the growth and physiology of fish [126,127,128]. The bioaccumulation of Cu in different organs of fish species is exhibited in Table 1. Several studies revealed that the liver is the main site that accumulates a significant proportion of Cu in comparison with the other organs [129,130,131]. Excess Cu in the fish diet reduced the fish appetite, thus negatively affecting the feed utilization and growth of fish [132]. Moreover, Cu toxicity not only resulted in deformed reproductive organs but also drastically reduced the GSI, fecundity, fertilization, and hatching rate of several fish species [18,21,133].

### 2.5. Mn

Mn is commonly found in a wide range of environments [134]. Mn was found to dissolve into water bodies through various anthropogenic activities [135]. Several factors, including fish species, age, and water quality, may vary Mn toxicity in fish [136]. It has been revealed that Mn toxicity declines with the increase in water hardness [134]. The bioaccumulation of Mn in the liver, gills, and muscles of *Argyrosomus japonicas* disturbed the metabolic process of carbohydrates and altered the ionic profile of blood plasma [137]. Mn affects the physiology of fish and sometimes exhibits fatal and lethal effects [134]. Mn exposure results in oxidative stress in *C. auratus* [138]. Mn results in many neurogenetical disorders by inducing the formation of free radicals and the inactivation of several enzymes associated with an antioxidant capacity [139]. Additionally, Mn damages the liver and induces cell apoptosis of grouper [140].

### 2.6. Ni

Ni is extensively used in different industrial activities and is considered a dominant contaminant of aquatic systems. Basically, Ni in aquatic ecosystems combines with other chemical compounds to form soluble salts that have the ability to adsorb onto other substances and cause several synergistic and antagonistic effects [141]. The severity of Ni toxicity depends on various factors such as Ni concentration, water quality, and the physiological state of organisms [142]. Several studies revealed that Ni accumulated in different organs of fish, especially in gills, and resulted in complexities in respiratory functions [143,144,145,146,147]. In addition, Ni was found to accumulate in the intestine of fish and disrupt the function of the intestine [148,149]. Ni alters the normal physiology and causes the death of several freshwater fish species [150]. Ni contamination induces several histological deformations of gills, including hyperplasia, hypertrophy, and fusion of gill lamellae in *Orechromis niloticus* [151]. Additionally, Ni toxicity hampers ion regulation [152,153,154] and induces oxidative stress in fish [155,156,157,158,159]. Two studies observed no significant impacts on fish growth [160,161]; however, they showed significant effects on the growth of pulmonate snails [162] and zebrafish [163].

### 2.7. Pb

Pb is a potent hazardous element that is bioaccumulated in aquatic organisms through water and feed [164]. Pb is bioaccumulated in different fish organs, including the liver, kidney, gills, spleen, and even the digestive system [165,166,167,168,169,170,171]. Pb significantly changes the blood parameters of fish [172,173,174,175,176]. Additionally, Pb toxicity results in a significant alteration in enzyme activity in blood plasma and the liver of fish that causes several pathologies in the cell membrane and shreds the liver cell [175,177,178,179]. Pb negatively affects the growth and feed utility of fish by reducing weight gain, specific growth rate, and feed intake [180,181,182,183]. Moreover, Pb results in poor reproductive performances such as low-quality sperm and ovum, reduced fertilization and hatching rate, low survival of embryo and larvae, etc. [17,20].

### 2.8. Zn

Zn is an essential micronutrient that plays a significant role in the growth and reproduction of fish [27,184,185]; however, an excess amount of Zn has several hazardous effects on fish [186]. Zn contamination in aquatic ecosystems is well established [187,188]. Liver and kidney tissues are the main sites for Zn bioaccumulation [189]. Zn toxicity negatively affects the growth [190,191,192], reproduction [22], homeostasis [193], feed intake [194,195,196], and bone formation of fish [197]. Zn toxicity induces ammonia excretion that results in poor water quality and stressful conditions for fish [191]. In addition, Zn toxicity damages the fish liver by increasing the activity of ALT and AST [198,199,200]. Moreover, high Zn levels significantly reduce the body protein and lipids of fish, which might result in the oxidation of protein and lipids, as well as low protein intake [201,202,203,204].

**Table 1:** Bioaccumulation of heavy metals in different tissues of fish.

Species	Doses	Exposure Time (Days)	Organs	Bioaccumulation Rate	References
As					
*Oreochromis niloticus*	806.5 and 772.1 µg/g	10	Gills	5.12 ± 0.61 μg/g	[78]
			Liver	9.51 ± 1.68 μg/g	
			Muscle	3.40 ± 0.24 μg/g	
		20	Gills	4.94 ± 4.62 μg/g	
			Liver	10.04 ± 2.99 μg/g	
			Muscle	3.74 ± 3.38 μg/g	
*Siganus fuscescens*	400 and 1500 μg/g	21/42	liver	63.3–91.3%	[77]
			Muscle	79.0–95.2%	
Cd					
*Oreochromis niloticus*	0, 0.1, and 1.0 mg/L	30	Gills	22.34–32.26 µg/g	[111]
			Liver	114.5–274.9 µg/g	
			Muscle	2.02–2.50 µg/g	
*Oreochromis niloticus*	0, 1.68, 3.36, and 5.03 mg/L	10	Gills	0.19–31.65 µg/g	[205]
			Liver	0.29 µg/g	
			Muscle	0.03 µg/g	
		20	Gills	0.28 µg/g	
			Liver	0.41–138.12 µg/g	
			Muscle	0.08–1.41 µg/g	
*Cyprinus carpio*	5 mg/L	32	Gills	6.23–6.94 μg/g	[110]
			Liver	4.82–5.64 μg/g	
			Kidney	4.31–5.32 μg/g	
*Oreochromis niloticus*	0.1 mg/L	30	Gills	23.18 µg/g	[129]
			Liver	114.5 µg/g	
			Muscle	2.02 µg/g	
	1.0 mg/L		Gills	32.26 µg/g	
			Liver	274.9 µg/g	
			Muscle	2.50 µg/g	
*Oncorhynchus mykiss*	1.5 mg/kg	36	Gills	0.20–0.30 μg/g	[206]
			Liver	0.29–0.37 μg/g	
			Carcass	0.19–0.32 μg/g	
	15 mg/kg		Gills	0.18–0.20 μg/g	
			Liver	0.36–0.40 μg/g	
			Carcass	0.28–1.18 μg/g	
	150 mg/kg		Gill	0.24–0.32 μg/g	
			Liver	0.40–0.93 μg/g	
			Carcass	0.37–1.67 μg/g	
	1500 mg/kg		Gills	0.54–1.77 μg/g	
			Liver	1.20–6.47 μg/g	
			Carcass	1.03–1.82 μg/g	
*Oreochromis niloticus*	0.0,1.68, 3.36, and 5.03 mg/L	30	Gills	0.32–61.73 µg/g	[205]
			Liver	0.96–181.61 µg/g	
			Muscle	0.12–2.16 µg/g	
*Oncorhynchus mykiss*	0.0 and 3.0 µg/L	30	Gill	0.72–6.48 µg /g	[207]
			Liver	1.29–3.87 µg /g	
			Kidney	0.47–9.40 µg /g	
Cr					
*Cyprinus carpio*	0.0, 3.41 mg/L	4	Gills	0.65–0.80 µg/g	[111]
			Intestine	0.50–0.60 µg/g	
			Muscles	0.40–0.45 µg/g	
			Skin	0.30–0.35 µg/g	
			Bone	0.60–0.60 µg/g	
*Carassius auratus*	4.00 mg/L	1	Gills	5.43 μg/g	[112]
			Intestine	3.9 μg/g	
			Skin	3.21 μg/g	
	6.00 mg/L	2	Gills	5.04 μg/g	
			Intestine	3.72 μg/g	
			Skin	3.18 μg/g	
	8.00 mg/L		Gills	4.69 μg/g	
			Intestine	3.63 μg/g	
			Skin	3.03 μg/g	
	12.00 mg/L		Gill	4.11 μg/g	
			Intestine	3.51 μg/g	
			Skin	1.98 μg/g	
*Oreochromis aureus*	0, 10, 15, 20, 25, and 30 mg/L	28	Gills	3.06–44.83 μg/g	[113]
			Skin	2.72–25.3 μg/g	
			Muscle	1.25–12.25 μg/g	
*Cirrhinus mrigala*	1.82 mg/L	7–28	Gills	16.54–48.74 μg/g	[109]
			Liver	27.52–87.33 μg/g	
			Kidney	21.23–97.33 μg/g	
			Muscle	12.23–48.64 μg/g	
	6.07 mg/L		Gills	19.82–36.83 μg/g	
			Liver	51.63–78.93 μg/g	
			Kidney	37.72–162.64 μg/g	
			Muscle	27.83–91.23 μg/g	
*Cyprinus carpio*	5 mg/L	32	Gills	2.25–3.56 μg/g	[110]
			Liver	2.66–4.27 μg/g	
			Kidney	2.773–3.233 μg/g	
Cu					
*Oreochromis* sp.	0.0, 0.50, 1.0, 3.0, and 5.0 mg/L		Gills	6.3–38.4 mg/kg	[131]
			Liver	19.4–136 mg/kg	
			Muscle	1.4–4.0 mg/kg	
*Sparus aurata*	0.0 and 0.1 mg/L	11	Gills	1.26–5.03 µg/g	[208]
			Liver	3.24–7.02 µg/g	
			Muscle	0.85–1.49 µg/g	
*Mystus vittatus*	0.0 and 5.98 mg/L	28	Gills	9.84–63.69 µg/g	[130]
			Liver	10.63–70.65 µg/g	
			Kidney	8.77–54.09 µg/g	
			Muscle	0.32–0.86 µg/g	
*Oreochromis niloticus*	0.0, 0.1, and 1.0 mg/L	30	Gills	7.02–40.67 µg/g	[129]
			Liver	12.31–618.6 µg/g	
			Muscle	1.49–62 µg/g	
*Oreochromis niloticus*	0.1 mg/L	30	Liver	589.5 µg/g	[129]
			Gills	27.52 µg/g	
			Muscle	4.54 µg/g	
	1.0 mg/L		Gills	40.67 µg/g	
			Liver	618.61 µg/g	
			Muscle	6.20 µg/g	
Mn					
*Cyprinus carpio*	0.0, 1.12, and 3.41 mg/L	4	Gills	0.49–0.93 µg/g	[111]
			Intestine	0.07–0.18 µg/g	
			Muscles	0.07–0.12 µg/g	
			Skin	0.04–0.1 µg/g	
			Bone	0.03–0.07 µg/g	
Ni					
*Cyprinus carpio*	5 mg/L	32	Gills	3.17–3.94 μg/g	[110]
			Liver	3.75–4.80 μg/g	
			Kidney	0.15–1.61 μg/g	
Pb					
*Clarias gariepinus*	0, 16, 32, and 48 mg/L	10	Gills	0.17–9.60 mg/100 g	[171]
			Liver	0.08–4.42 mg/100 g	
			Muscles	0.23–0.96 mg/100 g	
			Skin	0.09–1.14 mg/100 g	
		20	Gills	0.16–19.18 mg/100 g	
			Liver	0.12–5.54 mg/100 g	
			Muscles	0.19–1.27 mg/100 g	
			Skin	0.07–0.32 mg/100 g	
*Carassius auratus*	0.09, 0.15, 0.24, 0.3, 0.36, and 0.45 mg/L	28	Gills	0.00–0.71 mg/g	[169]
			Muscle	0.00–0.23 mg/g	
			Visceral	0.00–3.65 mg/g	
*Clarias gariepinus*	0.032, 0.052, and 0.160 mg/L	56	Gills	33.49 µg/g	[168]
			Liver	26.94 µg/g	
			Muscle	12.63 µg/g	
*Catla catla*	0.0, 1.0, 2.5, 5.0, 7.5, and 10.0 µg/L	42	Gills	4.71 µg/g	[209]
			Skin	4.92 μg/g	
			Eyes	4.51 µg/g	
			Liver	4.79 µg/g	
			Muscle	4.41 µg/g	
			Intestine	4.21 µg/g	
*Oreochromis niloticus*	0, 100, 400, and 800 μg/g	60	Liver	0.021–4.163 μg/g	[167]
			Stomach	0.025–11.68 μg/g	
			Intestine	0.021–31.75 μg/g	
*Cyprinus carpio*	5 mg/L	32	Gills	4.28–4.99 μg/g	[110]
			Liver	7.33–8.74 μg/g	
			kidney	6.33–6.94 μg/g	
*Oncorhynchus mykiss*	7, 77, and 520 µg/g	21	Gills	8.0 μg/g	[210]
			Intestine	17.8 µg/g	
			Liver	1.9 μg/g	
			Kidney	2.4 μg/g	
			Carcass	2.7 μg/g	
Zn					
*Oreochromis* sp.	0.0, 0.50, 1.0, 3.0, and 5.0 mg/L	4	Gills	49.5–98.1 mg/kg	[111]
			Liver	93.9–422.8 mg/kg	
			Muscle	11.5–30.8 mg/kg	
*Oreochromis* *Niloticus*	0.0, 3.5, and 7.0 mg/L	45	Gills	22.8–83.2 mg/L	[189]
			Liver	24.9–109.5 mg/L	
			kidney	24.6–93.5 mg/L	
			Muscles	9.5–20.2 mg/L	
*Sparus aurata*	0.0 and 0.1 mg/L	11	Gills	23.72–28.94 µg/g	[208]
			Liver	24.57–39.56 µg/g	
			Muscle	18.13–19.36 µg/g	
*Channa punctatus*	6.62 and 13.24 mg/L	45	Muscle	4.95–5.29 µg/g	[186]

**Table 2:** Heavy metals toxicity in fishes.

Species	Toxicity	References
As		
*Clarias batrachus*	Hematological: Serum protein level significantly declined	[211]
*Tilapia mossambica*	Hemato-biochemical: WBC, MCH, and MCHC levels increased while Hb, RBC, and PCV levels decreased significantly	[212]
*Oreochromis mossambicus*	Gill: Joined lamellae, hyperplasia, and necrosis noticed in epithelial cells Liver: Infiltrated macrophages, shrank and congested hepatic cells, hypertrophy, and vacuolation	[213]
*Clarias batrachus*	Kidney: Vacuoles, melanomacrophages	[86]
*Danio rerio*	Reproduction: Number of eggs, spawns, and hatching rate significantly declined	[92]
Cd		
*Catla catla*	Gill: Atrophy, telangiectasia, and necrosis Liver: Degenerated, congested, and hemorrhagic hepatocytes Kidney: Atrophic glomerulus, degenerated and necrotic renal tubes, and melanomacrophages	[214]
*Mystus seenghala*	Poor growth as well as feed utility	[215]
*Pelteobagrus fulvidraco*	Significantly lowered weight gain and specific growth rate	[216]
*Clarias gariepinus*	Hemato-biochemical: AST, ALP, ALT, Cort, Glu, and MCH increased while CK, TLC, and MCV decreased	[217]
*Channa striata*	Hemato-biochemical: HDL, LDL, TP, AST, and ALT increased while Glu level decreased	[218]
*Cyprinus carpio*	Gill: Spiked and fused lamellae, club-shaped epithelial filaments in lamellae Liver: Hepatic cells with ruptured veins and vacuoles	[219]
*Cyprinus carpio*	Gill: Fused gill lamellae, widened vessel, hyperemia, and hyperplasia in epithelial cells	[170]
*Labeo rohita*	Hemato-biochemical: WBC level increased significantly while RBC and Hct decreased significantly	[220]
Cr		
*Pangasianodon hypophthalmus*	Blood cells: Caused erythrocytic cellular and nuclear complexities Gill, liver & kidney: Histopathologies observed in gills, liver, and kidney Hematology: WBCs and blood glucose levels increased	[1]
*Oryzias melastigma*	Liver: Vacuoles, pyknotic cells, and abnormal nucleus observed in hepatic cells	[124]
*Oreochromis niloticus*	Weight gain and specific growth rate reduced	[221]
*Pangasianodon hypophthalmus*	Blood cells: Different anomalies observed in erythrocytes Hematology: RBC, Hb, and PCV decreased significantly	[117]
*Anabas testudineus*	Gill: Fused, hemorrhaged gill lamellae Kidney; Edema, interstitial hemorrhage, and degenerated renal tubules found in renal tissues	[222]
*Channa punctatus*	Hemato-biochemical: Albumin, triglyceride, HDL, and VLDL levels in serum decreased	[223]
*Oryzias latipes*	Reproduction: GSI and fecundity significantly lowered Frequencies of immature oocytes and spermatozoa increased in gonad	[123]
Cu		
*Cyprinus carpio*	Significantly reduced the growth and feed utilization indices	[224]
*Channa gachua*	Liver: Vacuoles in the cytoplasm and stroma, degenerated nuclei	[225]
*Poecilia reticulata*	Reproduction: Poor reproductive performances, increased parturition period, highest mortality of larvae	[21]
*Oryzias melastigma*	Improper skeletal structures, anomalies in the vascular system, lower pigmentation of embryo	[226]
*Leuciscus idus*	Yolk sac malformation, lower body length, and perimeter, curve vertebrae	[26]
Pb		
*Myoxocephalus scorpius*	Gill: Gill lamellae fused, hyperplastic epithelial cells, synechia, and telangiectasia Liver; Hepatosis, necrosis, granuloma, and neoplasm in hepatocytes	[227]
*Mugil cephalus*	Hemato-biochemical: Glucose and malondialdehyde levels in the blood increased	[228]
*Chanos chanos*	Growth significantly reduced with the increase of Pb concentration	[183]
*Cyprinus carpio*	Gill: Spiked and fused secondary lamellae, club-shaped filaments epithelium	[219]
*Carassius gibelio*	Poor reproductive performance	[17]
*Labeo rohita*	Hemato-biochemical: RBCs and Hct decreased while WBCs increased	[220]

## 3. Bioremediation of Heavy Metal Toxicity in Fishes

**Figure 2:**
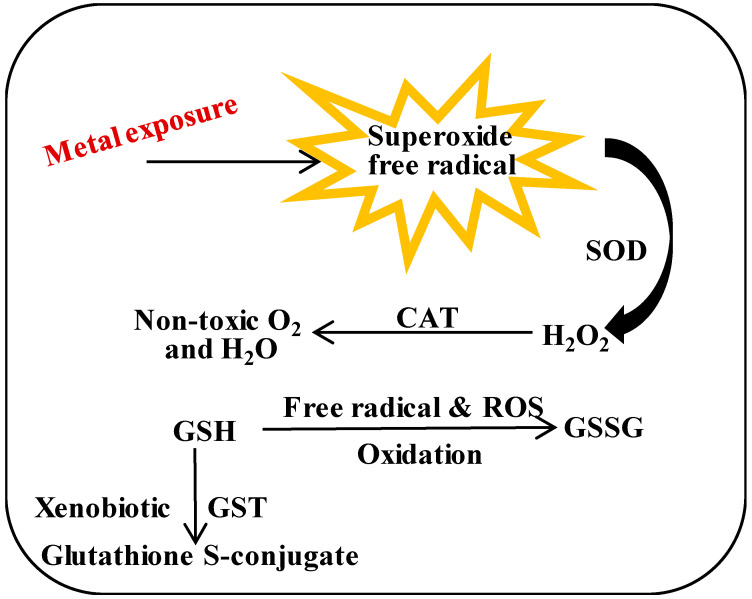
Heavy metals detoxification mechanism; SOD, superoxide dismutase; CAT, catalase; GST, glutathione S transferase; GSH, glutathione; GSSG, glutathione disulphide; ROS, reactive oxygen species.

Bioremediation is a convenient and ecofriendly option that can be used to restore the contaminated environment by removing toxic metals from the environment [224,229]. Bioremediation of toxicants can be done by adsorption [230,231,232], physio-biochemical mechanisms [233,234,235,236], and molecular mechanisms [237,238,239]. Several enzymes (superoxide dismutase, SOD; catalase, CAT; glutathione S transferase, GST) and nonenzymatic compounds (reduced glutathione, GSH) play a key role in sustaining the ROS balance by detoxification (Figure 2). SOD has the capability to convert superoxide radicals to hydrogen peroxide radicals that transform into nontoxic oxygen and water through the activity of CAT enzymes [240]. On the other hand, GST detoxifies the toxicants by catalyzing electrophiles to GSH. Moreover, GSH converts into glutathione disulfide through the nonenzymatic oxidation of electrophilic compounds including free radicals and ROS [223].

Phytoremediation is a popular bioremediation technique in which various plants and microbes are used to reduce pollutants from the aquatic environment [4]. Microbial enzymes play a key role in converting toxic contaminants to safe ones by altering the chemical structure of contaminants. Some *Lactobacillus* spp. efficiently remediate heavy metals by converting the environment more acidic in nature [241,242] and through biosorption means or by forming bonds between heavy metals and their cell components [243]. Several mechanisms of microorganisms that make them resistant to heavy metals include extracellular sequestration [244,245], intracellular sequestration [246,247], reduction of heavy metal ions by the microbial cell [248], and extracellular barriers [249,250]. A wide range of microbes, such as bacteria, fungi, and algal species, have been used to detoxify heavy metals [251,252] and keep the environment clean; they are listed in Table 3. In addition to natural microbes, several genetically improved engineered microorganisms, especially surface-engineered microorganisms, were developed to use in the remediation process of target-specific heavy metals [253]. Several studies reported that the capabilities of genetically engineered microbes are greater than natural microbes for removing organic compounds, including heavy metals, under natural environmental systems [254,255]. Several engineering aspects, including single-gene edition, metabolic pathway modification, and alteration of gene sequences (coding and controlling), are successfully employed to modify the genetic makeup of microorganisms and transform them into engineered microorganisms [60], which more efficiently eliminates several heavy metals such as Ni, Hg, Cd, Fe, As, and Cu [256,257,258]. Additionally, the application of advanced engineering approaches (genomics, metagenomics, proteomics, metabolomics, and transcriptomics) has produced genetically modified microbes that play a crucial role in the bioremediation of several heavy metals [63,259]. The application of genetically modified *Pseudomonas putida* and *Escherichia coli* strain M109 has successfully removed Hg from contaminated sites [260]. The insertion of mer genes in *Deinococcus geothemalis* [4] and *Cupriavidus metallidurans* strain MSR33 [4,261] has been found to efficiently reduce Hg. Moreover, transporters in microbial membranes significantly improves the bioremediation of heavy metals from the environment [60,61]. It has been revealed that dietary *Lactobacillus plantarum* alleviated the toxicity caused by aluminum (Al) in tilapia [262]. The application of *Spirulina platensis* significantly alters the negative effects of As toxicity in *Oryzias latipes* [90]. Additionally, the provision of probiotics in the diet has been found to reverse the negative effects of Cd on the growth and hematology of *Oreochromis niloticus* [263]. However, EDTA significantly reduced the body Cd level and, thus, improved the blood profile of *Clarias gariepinus* [264]. Pomegranate peel and *Lactococcus lactis* have shown positive results in remediating toxicity resulting from Hg in *C. gariepinus* [265,266]. Probiotic supplementation (*L. reuteri*) was found to effectively alter the negative effects of Pb in *Cyprinus carpio* [182,267].

### 3.1. Application of Bacteria as Bioremediator

Bacteria play an important role due to having some special features such as their size, distribution, and capability to grow in controlled and resilient environments [268]. It has been reported that 70% and 75% Cd are removed by the application of *P. aeruginosa* and *Alcaligenes faecalis*, respectively [269], while *Bacillus pumilus* and *Brevibacterium iodinium* are able to remove Pb up to 87% and 88%, respectively. Another study revealed that *B. cereus* had the ability to remove 72% Cr [251]. *Micrococcus luteus* can remove a significant amount of Pb [270]. It has been stated that the highest quantity of Pb, Cr, and Cd were removed by *B. megaterium*, *Aspergillus niger*, and *B. subtilis*, respectively [271]. *Desulfovibrio desulfuricans* was found to effectively reduce 99.8% Cr, 98.2% Cu, and 90.1% Ni [252]. It has been reported that mixtures of several bacterial species efficiently removed Cr, Zn, Cd, Pb, Cu, and Co [272].

### 3.2. Application of Fungi as Bioremediator

Several fungal species with great capability of metal uptake and recovery are widely used to remediate toxic metals [273,274]. Several studies reported that both live and dead cells of fungal species actively adsorb metals [275,276]. It has been reported that *Aspergillus* sp. efficiently removed 85% of Cr [277]. It has been revealed that dead cells of *Aspergillus niger*, *Rhizopus oryzae*, *Saccharomyces cerevisiae*, and *Penicillium chrysogenum* are suitable for transforming toxic Cr into a less toxic form [278]. Another fungal species, *Coprinopsis atramentaria*, is considered an important metal accumulator [279]. *Candida sphaerica* was found to significantly reduce metal loads by creating biosurfactants [280,281]. Moreover, *Hansenula polymorpha*, *Saccharomyces cerevisiae*, *Yarrowia lipolytica*, *Rhodotorula pilimanae*, *Pichia guilliermondii*, and *Rhodotorula mucilage* were found to effectively convert toxic Cr to a less toxic state [282,283,284].

### 3.3. Application of Algae as Bioremediator

Algae have a high biosorption capacity and, hence, are used as biosorbents to remove toxic heavy metals [285]. Several algae and cyanobacterial species have the capability to either remove or degrade toxic metals [208]. This degradation of toxic metals by algae may be attributed to the high photosynthetic capacity of algae, resulting in the availability of significant amounts of dissolved oxygen in aquatic systems that caused an aerobic breakdown of several organic compounds, including heavy metals. Heavy metals, as well as other toxic compounds, may be degraded, detoxified, and transformed through several enzymatic and metabolic activities of algae in their metabolism [286]. Moreover, the algal cell wall is composed of several essential functional groups (fucoidan, alginate) that play a significant role in the removal of toxic heavy metals through a biosorption mechanism [287,288]. Additionally, algae can bind heavy metals through the employment of several binding approaches (extracellular and intracellular), including chelation, complexation, and physical adsorption to lessen the associated toxicity [289]. In addition, algae can play a very significant role in the detoxification of metals through their ability to synthesize class III metallothioneins (phytochelatins) that are synthesized by phytochelatin synthase enzymes that require post-translational activation by heavy metals [290,291,292]. The algal surface contains various chemical substances such as hydroxyl, carboxyl, phosphate, and amide that act as binding sites for the metals [293]. Death cells of another important algal species, *Chlorella vulgaris*, have been established as an efficient remover of Cd, Cu, and Pb [294].

**Table 3:** Bioremediation of heavy metals toxicity in different fishes.

Species	Doses	Exposure Time (Days)	Bioremediation	References
Al				
*Oreochromis niloticus*	Al (2.73 mg/L)	28	Increased Al level in organs and reduced RBCs, WBC, GB, HCT, MCV, MCH, SOD, GPx, CAT, TAO, and liver injured	[262]
	Al (2.73 mg/L) + *L. plantarum* (10^8^ cfu/g)		Enhanced growth performance, decreased mortality, and Al levels alleviated the alteration of hepatic oxidative stress, histopathology, and hematological parameters	
As				
*Oryzias latipes*	As (7, 10 ppm)	15	Increased apoptotic, formation of MN, RBC, and DNA damage	[90]
	As (7, 10 ppm) + *Spirulina platensis* (200 mg/L)		Mitigated As toxicity and repaired DNA damage	
*Pangasianodon hypophthalmus*	As (2.68 mg/L) +T (34 °C)	90	Increased stress responses and decreased growth efficiency	[295]
	Se-NPs (0.5 mg/kg diet) + RF-(5/10/15 mg/kg) + As+ T			
			Enhanced growth performance, antioxidative status, immunity of the fish, and reduced stress biomarkers.	
Cd				
*Oreochromis niloticus*	Cd (0.3, 0.6 ppm)	30	Reduced length of testicular cell size	[229]
	Cd (0.3/0.6 ppm) + P (200 mg/kg)		Significantly increased length of testicular cell size	
	Cd (0.3 /0.6 ppm) + Vit-C (200 mg/kg)		Significantly increased length of testicular cell size	
	Cd (0.3/0.6 ppm) + P (200 mg/kg) + Vit-C (200 mg/kg)		Significantly increased length of testicular cell size	
*Oreochromis niloticus*	Cd (5 mg/L)	45	Elevated levels of antioxidants gene transcript levels, GST-⍺1, GPx1, and MT caused oxicopathic lesion	[296]
	Cd (5 mg/L) + Vit-C (500 mg/kg)		Prevented the Cd-induced toxicopathic lesion and decreased hepatoxicity	
*Oreochromis niloticus*	Cd (1 mg/L)	28	Reduced growth rate, altered hemato-biochemical parameters, increased mortality, and reduced gut microbial diversity	[263]
	Cd (1 mg/L) + *Lactobacillus plantarum* (10^8^ CFU/g)		Improved growth performance, decreased mortality, and Cd level reversed alteration of hemato-biochemical parameters in blood	
*Oreochromis niloticus*	Cd (10 ppm)	15 and 45	Reduced RBCs, Hb, Hct, MCH, and TP and increased ALT, ACP, AHT, and LP	[297]
	Cd (10 ppm) + EDTA (0.2 or 0.3 g/L)		Reduced Cd from the fish body and enhanced growth rate and hemato-biochemical parameters	
*Clarias gariepinus*	Cd (12 ppm)	45	Reduced RBCs, Hb, Hct, MCV, MCH, and MCHC	[264]
	Cd (12 ppm) + EDTA (0.3 mg/L)		Eliminated Cd from the fish body, thus improving hemato-biochemical parameters	
*Carassius gibelio*	Cd (10 ppm)	21	Increased Cd level in kidney and intestine and reduced Fe, Zn, and Cu	[298]
	Cd (10 ppm) + Zeolite (4 mg/L)		Reduced Cd level from organs and mitigated the antagonistic impact of Cd on some minerals such as Fe, Zn, and Cu	
*Oreochromis niloticus*	Cadmium (10 ppm)	45	Reduced RBCs, HB, HCt, MCH, and MCHC and increased MCV	[299]
	Cadmium (10 ppm)+ Fulvic acid0.3 g/L		Regulated RBCs, HB HCt, MCV, MCH, and MCHC to normal level	
*Oreochromis mossambicus*	Cd (6 ppm)	15	Decreased RNA:DNA ratio and reduced growth	[155]
	Cd (6 ppm) + Zeolite (4.0 g/L)		Increased RNA:DNA ratio 6 to 10 times in liver, muscle, and gills and enhanced growth performance	
*Ctenopharyngodon idella*	Cd (5 mg/L)	15	Increased Cd level and caused structural damage in the organs	[300]
	Cd (5 mg/L) + *L. gibba* L (1 g/L)		Decreased Cd residue in liver and muscle	
	Cd (5 mg/L) + *S. platensis* (5 mg/L)		Significantly decreased Cd residue in liver and muscle	
	Cd (5 mg/L) + *L. gibba* (1 g/L) + *S. platensis* (5 mg/L)		Remediated the degenerative action of Cd in fish	
Cu				
*Cyprinus carpio*	Cu (0.1 mg/L)	14	Increased Cu level in fish	[224]
	Cu (0.1 mg/L) + *Stenotrophomonas maltophilia* (10^8^ cfu/mL)		Greatly reduced Cu accumulation from fish	
*Oreochromis mossambicus*	Cu (2.14 or 4.27 mg/L)	180	Increased Cu level in fish tissue	[301]
	Cu (2.14 mg/L) + Zeolite (2 g/L)		Removed Cu content from fish	
*Oreochromis mossambicu*	Cu (4.27 ppm)	28	Reduced RBCs, Hb, and Ht value, O_2_ carrying capacity of the blood and increased TLC and ESR	[302]
	Cu (4.27 ppm) + EDTA (0.125/0.25/0.50/1.0) g/L		Increased RBCs, HB, and HCt value and greatly reduced Cu accumulation	
Hg				
*Clarias gariepinus*	Hg (0.13 ppm)	60	Increased AST, ALT, urea, and creatinine levels and reduced GH, GSH, and MDA	[265]
	Hg (0.13 ppm) + PPW (0.3 g/L)		Reduced urea and creatinine levels	
	Hg (0.13 ppm) + PPD (1 or 2 g/L)		Increased RBCs, Hb, PCV, lysozyme, and antiprotease activity and reduced Hg level	
	Hg (0.13 ppm)	60	Reduced RBCs, Hb, WBC, globulin, GSH, and MDA	[266]
	Hg (0.13 ppm) + *Lactococcus* *lactis* (10^9^/10^10^ cfu/g)		Increased RBC, WBC, lysozyme, antiprotease activity, nitric-oxide, GSH, MDA, TP, albumin, and globulin	
*Oreochromius niloticus*	Hg (75 μg/L)	15	Reduced RBC, Hb, Hct, AST, ALT, and ALP	[303]
	Hg (75 μg/L) + *S. platensis* (5/10 mg/L)		Reduced Hg level and improved the hematological parameters (RBCs, Hb, and Hct)	
Ni				
*Cyprinus carpio*	Ni (1.0 mg/L)	14	Enhanced Ni concentration in fish	[224]
	Ni (1.0 mg/L) + *Stenotrophomonas maltophilia* (10^8^ cfu/mL)		Greatly reduced Ni level from fish	
Pb				
*Cyprinus carpio*	Pb (50 or 100 mg/L) +*Lactobacilli ruteri*		Exhibited the best Pb-binding ability, thus removing Pb level	[267]
*Cyprinus carpio*	Pb (1 mg/L)	42	Increased mortality and decreased growth rate, RBCs, WBC	[182]
	Pb (1 mg/L) + *L. reuteri* (10^8^ cfu/g)		Reduced mortality and Pb accumulation and improved growth performance, and immune response of fish	
*Oreochromis niloticus*	Pb (10 mg/kg)	60	Reduced GH Level and growth performance	[304]
	Pb (10 mg/kg) + VE (300 mg/kg)		Reduced Pb accumulation in tissue and significantly increased serum GH level	
*Oreochromis niloticus*	Pb (81.53 mg/kg)	70	Reduced PCV, RBCs, WBC, and lymphocytes and increased lipid peroxidation level (malondialdehyde)	[305]
	Pb (81.53 mg/kg) + Vitamin-E (200 mg/kg) + Selenium (40 mg/kg)		Increased blood parameters prevented cell damage by reducing malondialdehyde	
*Cyprinus carpio*	Pb (0.017 mg/L)	5	Caused lipid peroxidation and altered antioxidant enzymes SOD, CAT, proteins, glucose, glycogen, and amino acids in organs	[306]
	Pb (0.017 mg/L) + Spirulina (500 mg/fish)		Reduced Pb toxicity and enhanced SOD, and CAT activity in the liver and gills, thereby diminishing lipid peroxidation	
*Oreochromis niloticus*	Pb (1 mg/L)	28	Reduced Final BW, ADG, SGR, and GSH and increased FCR and NAs	[181]
	Pb (1 mg/L) + *Lactobacillus plantarum* (10^8^ CFU/g)		Increased growth performance and reduced NAs, mortality rate, and Pb level in fish organs	
*Heteropneusts fossilis*	Pb (10 mg/L)	180	Reduced protein content in all tissue	[307]
	Pb (10 mg/L) + Chabazite (10 mg/L)		Increased protein content, thus improving fish quality	
Metal (Zn +Pb + Cd + Cu)				
*Oreochromis niloticus*	M (Cd + Cu + Pb + Zn) (5 mg/L)	5 and 7	Increased frequencies of MN, BN, other NAs, and MAE and altered erythrocytes	[308]
	M (5 mg/L) + Se (75 μg/kg) + Vit-A + E+C (300 μg + 10 mg + 60 mg/kg)		Reduced NAs and MAE, thus aiding in cell division in fish	

P—probiotic, RBC–red blood cell, WBC—white blood cell, Hb—hemoglobin, HCt—hematocrit, PCV—packed cell volume, MCV—mean corpuscular volume, MCH—mean cell hemoglobin, AST—aspartate amino transferase, ALT—alanine amino transferase, MCHC—mean cell hemoglobin concentration, MN—micronuclei, GB—globulin, LP—total lipid, TP—total protein, FCR—feed conversion ratio, NAs—nuclear abnormalities, MAE—morphologically altered erythrocytes, SGR—specific growth rate, BW—body weight, BN—binuclear, SOD—superoxide dismutase, CAT—catalase, GPx—glutathione peroxidase, TLC—total leucocyte count, ESR—erythrocyte sedimentation rate, GSH—glutathione, MDA—malondialdehyde, TAO—total antioxidant, ADG—average daily gain, ALP—alkaline phosphatase, MT—metallothionein, GST-α1—glutathione S-transferase, GH—growth hormone, Se-NPs—selenium nanoparticles, and RF—Riboflavin.

## 4. Conclusions and Recommendations

The discharge of toxic heavy metals without proper treatment from various industries is adversely deteriorating aquatic ecosystems. As a result, toxic heavy metals from this contaminated environment have bioaccumulated in several important organs of fish and disturbed their normal functions. The bioaccumulation of these toxic metals has severely affected the normal physiology of fish, reducing the growth and reproduction of fish. Bioremediation has great potentiality to reshape the existing contaminations of aquatic systems in a sustainable approach. Additionally, bioremediation improves fish health by altering the toxic effects of several heavy metals. It is not only beneficial for aquatic organisms but also improves the productivity of aquatic ecosystems. By efficient application of this bioremediation process, we can significantly recycle the water that reduces wastage of water, and degradation of organic matter lowers the pathogenic organisms that enhance the biosecurity of our ecosystems. In parallel with the current practices of bioremediation, genetically engineered microorganisms (GEM) should be introduced in the future to increase the efficiency of bioremediation techniques to mitigate adverse heavy metal contamination. In this case, public acceptance of GEM and the safety of the environment should be taken into consideration.

## Data Availability

The data that support the outcomes of this study are available on request from the corresponding author [M.S.].
